# COVID-19 Complicated by Spontaneous Pneumothorax

**DOI:** 10.7759/cureus.9104

**Published:** 2020-07-09

**Authors:** Taha Mallick, Anant Dinesh, Ryan Engdahl, Mario Sabado

**Affiliations:** 1 Surgery, Harlem Hospital Center, New York, USA; 2 Surgery, Columbia University College of Physicians and Surgeons, Harlem Hospital Center, New York, USA; 3 Thoracic and Vascular Surgery, Woodhull Medical Center, New York, USA

**Keywords:** covid-19, pneumothorax, neutrophils, chest tube, c reactive protein

## Abstract

Over the last few months, the coronavirus disease 2019 (COVID-19) pandemic has created overwhelming challenges for physicians across the world. While much has been described in the literature about lung infiltrates and respiratory failure associated with severe acute respiratory syndrome coronavirus 2 (SARS-CoV-2), pneumothorax remains a relatively rare presentation with current literature indicating a rate of one percent. We describe a case series of three patients each of whom tested positive for SARS-CoV-2 on reverse-transcriptase polymerase chain reaction testing of nasopharyngeal swab specimens and presented with pneumothorax. These patients were treated at the New York City Health and Hospitals (NYC H+H) system, a network of eleven hospitals in four different boroughs of New York City. None of these patients had a history of lung disease and one patient was a previous smoker. One out of three patients died. Inflammatory markers were noted to be elevated in each of these patients to levels that have been associated with severe COVID-19 infection. CT scans in these patients showed bilateral air space disease consistent with COVID-19 pneumonia and pneumothorax with other features including pneumomediastinum, subcutaneous emphysema, and pneumatoceles. This may indicate the underlying pathogenesis of pneumothorax in these patients to involve inflammation-induced pulmonary parenchymal injury and necrosis with subsequent development of air leaks into the pleural cavity, a mechanism similar to that noted in patients during the severe acute respiratory syndrome (SARS) outbreak in 2003. Conservative management with chest tube drainage or observation was adequate for two of three patients while one patient developed multi-organ system dysfunction and eventual death.

## Introduction

This article describes the clinical course of three patients diagnosed with coronavirus disease (COVID-19) infection who presented with spontaneous pneumothorax which is a relatively rare presentation of COVID-19 pneumonia. A single case series from China found pneumothorax as a presentation of COVID-19 in one out of ninety-nine patients [1]. Previous experience with severe acute respiratory syndrome (SARS) patients has indicated that patients most at risk of pneumothorax are those with more severe clinical courses and higher neutrophil counts and that a prolonged duration of lung inflammation may be needed for the development of pneumothorax [2]. Recent literature published on COVID-19 pneumonia has indicated that elevated levels of certain inflammatory markers are more likely to be associated with severe disease as defined by the fifth edition diagnosis and treatment program of 2019 novel coronavirus pneumonia issued by the National Health Commission of the People’s Republic of China [3]. We reviewed the elevations of these inflammatory markers during the course of our patients’ hospitalizations in this series as well as the duration and course of the disease. Using evidence from currently available literature we then proceed to explore possible similarities in the pathogenesis of spontaneous pneumothorax in certain patients infected with severe acute respiratory syndrome coronavirus 2 (SARS-CoV-2) and the SARS virus, both of which belong to the same *Coronaviridae* family.

## Case presentation

Case 1

**Figure 1:**
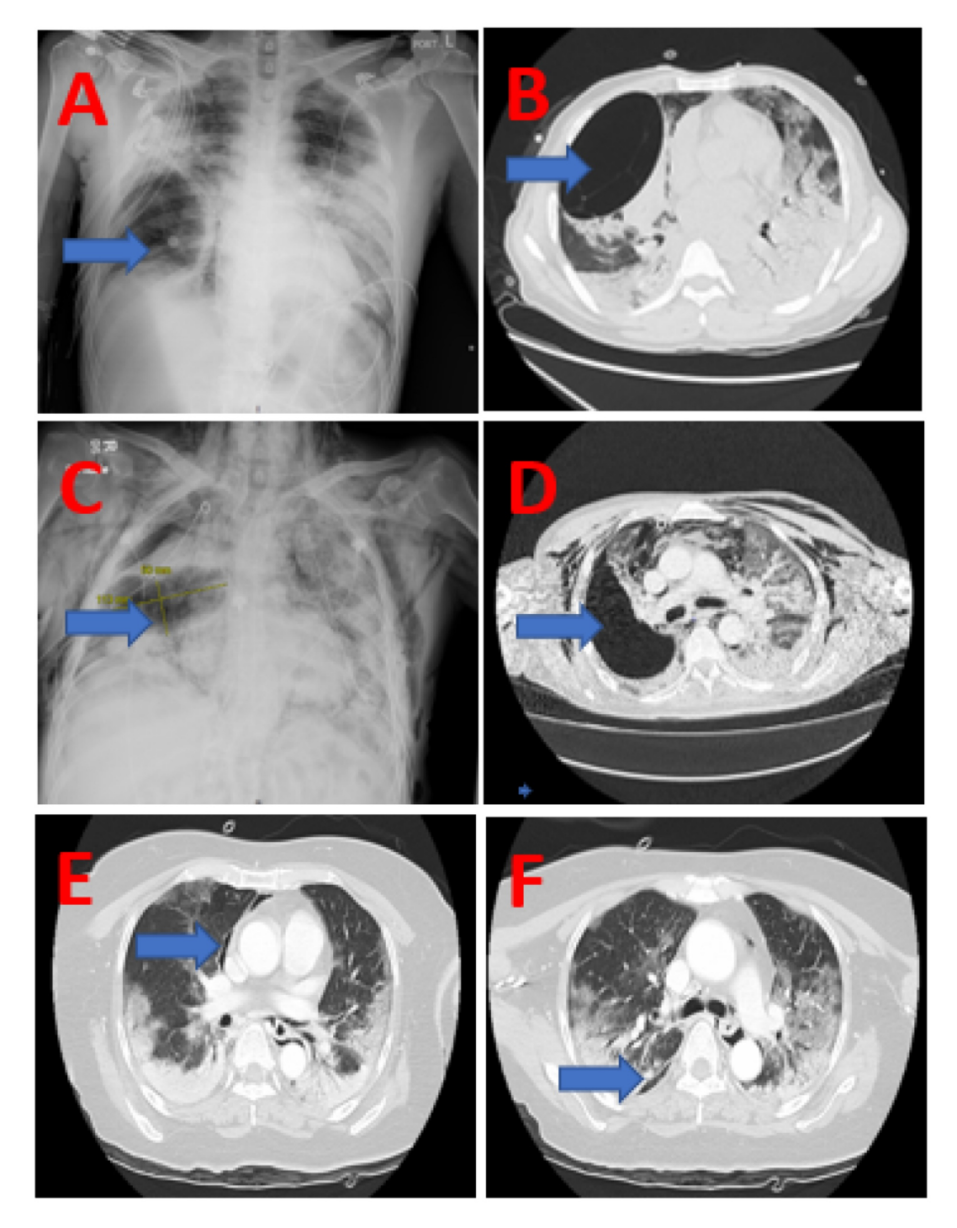
Radiologic findings in patients with COVID-19 and pneumothoraces (A) Case 1 with chest radiograph showing extensive bilateral airspace disease, right pigtail catheter placement and right pneumatocele (arrow). (B) Case 1 with confirmation of findings in (A) on CT scan including pneumatocele (arrow). (C) Case 2 with chest radiograph showing extensive bilateral airspace disease, bilateral chest tube placement and right pneumatocele (arrow). (D) Case 2 with confirmation of findings in (C) on CT scan including right pneumatocele (arrow). (E) Case 3 with CT scan showing evidence of pneumomediastinum along with bilateral air space disease. (F) Case 3 with CT scan showing evidence of small right upper lobe pneumothorax along with bilateral air space disease (arrow).

A 40-year-old male who was a previous smoker with no other notable past medical history, presented with one week of fevers, chills, and non-productive cough and a right pneumothorax on chest radiograph. On presentation, he was saturating 79% on room air which improved to 86% on the non-rebreather mask at a flow rate of 12 liters per minute. A pigtail catheter was placed and subsequent chest radiographs showed the lung to be adequately re-expanded and a 10 cm curvilinear space in addition to bilateral lung infiltrates and air bronchograms (Figure 1A). The patient was admitted and tested positive for COVID-19 with reverse transcription-polymerase chain reaction (RT-PCR) evaluation of a nasopharyngeal swab specimen. A chest CT scan demonstrated a large complex pneumatocele in the right middle lobe corresponding to the curvilinear space seen on previous chest radiographs (Figure 1B). Extensive bilateral airspace disease was also noted on radiologic studies consistent with COVID-19 pneumonia. The patient had a complicated clinical course with the development of multiorgan failure and eventual death on admission day 60.

Case 2

A 68-year-old male non-smoker with no notable past medical history presented with two weeks of cough, chest tightness, and vomiting. Initial oxygen saturation was 90% on room air and improved to 95% with facemask at a flow rate of 8 L/min. A chest radiograph showed bilateral infiltrates and he was admitted to the hospital where he tested positive for COVID-19. His oxygen requirements were weaned down and he was transferred to a lower acuity facility. However, nine days after discharge the patient returned to the emergency room with worsening respiratory distress. His O_2_ saturation was 70% on room air and improved to 90% with a non-rebreather mask at a flow rate of 15 L/min. A chest radiograph showed a large right pneumothorax along with a smaller left pneumothorax, pneumomediastinum, and subcutaneous emphysema. Bilateral chest tubes were placed. A subsequent radiograph showed bilateral lung re-expansion but with a right-sided ovoid lucency measuring 11.3 x 5 cm suggestive of a pneumatocele (Figure 1C). Extensive bilateral airspace disease was also noted in these radiologic studies. The patient was admitted and transferred to one of the several critical care units in the hospital. A chest CT scan confirmed the presence of an ovoid 12 x 4.8 cm air containing structure in the parenchyma of the right lower lobe probably representing a pneumatocele (Figure 1D). The left chest tube was removed on day five of admission while the right chest tube was removed on day 17. His oxygen requirements were weaned down during admission and he was discharged on day 25 on 3 L/min of oxygen.

Case 3

A 58-year-old female with a past medical history of hypertension and no known smoking history was brought in to the emergency room after being found at home with altered mental status. In the emergency room, she required 15 L/min of oxygen by non-rebreather mask to maintain an oxygen saturation of 90%. A CT scan of her head did not show intracranial abnormalities but did show retropharyngeal gas. Further imaging showed extensive bilateral pulmonary infiltrates along with pneumomediastinum (Figure 1E), subcutaneous emphysema, and a small right upper lobe pneumothorax (Figure 1F). Also noted were right pulmonary artery thrombosis and left renal vein thrombosis. This patient also subsequently tested positive for COVID-19. Her pneumothorax was managed without pleural cavity decompression and her oxygen requirement was weaned down to 2-3 liters per minute at which time she was discharged home on day 21 of admission. Her thromboses were managed with anti-coagulation followed by placement of an inferior vena cava (IVC) filter due to gastrointestinal (GI) bleeding.

## Discussion

In our experience with COVID-19 patients, we have noticed several cases of pneumothorax, pneumomediastinum, and subcutaneous emphysema in mechanically ventilated patients, which may be attributable to barotrauma. However, our patients presented in this case report did not require mechanical ventilation. None of our patients had a previous history of chronic obstructive pulmonary disease (COPD) or other lung diseases, and only one case had a previous smoking history. We, therefore, think that an alternate explanation for the development of these pneumothoraces should be sought. 

In trying to understand spontaneous pneumothorax in COVID-19 patients better, we draw on previous experience from SARS patients with spontaneous pneumothorax described in the literature [2]. Among six patients reported with SARS and spontaneous pneumothorax, notable features included a severe clinical course, higher mean neutrophil counts relative to comparable subsets of patients, and development of pneumothorax 14-37 days after hospital admission. The authors indicated that high levels of severe inflammation over a certain amount of time may cause pulmonary parenchymal injury with the development of pneumothorax and/or pneumomediastinum. Also noted in this study is a 1.7% incidence of spontaneous pneumothorax in SARS patients compared to one percent incidence for COVID-19 patients [1].

A recently published article showed several correlations between lab values and severity of COVID-19 infection as established by the National Health Commission of the People’s Republic of China in their fifth edition diagnosis and treatment program of 2019 novel coronavirus pneumonia [3]. In our case series of three patients with spontaneous pneumothoraces, each patient shows multiple lab values associated with severe infection (Table 1). Case one and two presented with a disease duration of at least one week while in case three duration of illness was unknown. We feel that the above features indicative of severe inflammation over a sustained period likely contributed to the pulmonary parenchymal injury in these patients with the eventual development of airleaks leading to pneumothorax and pneumomediastinum. This is very similar to what was seen in SARS patients. A point worthy of mention is that of the Macklin effect described as pneumomediastinum secondary to alveolar rupture due to an increase in intra-alveolar pressure [4]. This is plausible in COVID-19 patients due to coughing, which is known to increase intra-alveolar pressure. However, the Macklin effect has not been described to cause pneumothorax or pneumatoceles.

**Table 1:** Epidemiologic and clinical features of COVID-19 patients with spontaneous pneumothoraces *The patient was admitted with COVID-19 and discharged nine days prior to returning to the emergency department (ED) with bilateral pneumothorax. The patient was not intubated for COVID-19 at any time during either admission. WBC - white blood cell; NRB - non-rebreather mask; CRP - C-reactive protein; LDH - lactate dehydrogenase

Variables	Case 1	Case 2*	Case 3
Age (years)	40	68	58
Sex	Male	Male	Female
Duration of symptoms	1 week	4 weeks	Unknown
Smoking history	Previous smoker	None	None
Underlying lung disease	No	No	No
Pneumothorax	Unilateral, right	Bilateral	Unilateral, right
Associated findings	Pneumatocele (right)	Pneumatocele (right), pneumomediastinum, subcutaneous emphysema	Pneumomediastinum, subcutaneous emphysema
O_2_ saturation and O_2_ support at time of pneumothorax	79% on room air; 86% on 12L NRB	70% on room air; 90% on 15L NRB	90% on 15L NRB
Highest WBC count (x 10^9^/L)	25.98	19.01	20.18
Highest neutrophil percentage	93.3	85	92.7
Highest neutrophil count (x 10^9^/L)	22.41	15.71	19.44
Lowest lymphocyte percentage	2.6	6.7	4.4
Lowest lymphocyte count (x 10^9^/L)	0.39	0.75	0.43
Highest CRP (mg/L)	>300	191.97	119.9
Highest ferritin (ng/ml)	4929	1232	2086
Highest LDH (U/L)	1042	800	1098
Highest D-dimer (ng/ml)	9461	3064	52,493
Pleural cavity decompression	Yes	Yes	No
Ventilator required	Yes	No	No
Patient outcome	Critical, multiorgan failure with death on day 60	Discharged on day 25 with both chest tubes removed	Discharged on day 21 with spontaneous resolution of pneumothorax

Also notable is that two of our three patients harbored pneumatoceles. Again it seems likely that the same prolonged inflammation, which leads to the development of pneumothorax and pneumomediastinum may have led to the development of pneumatoceles through wall destruction of neighboring alveoli and airspaces. We also noted that in case two, chest radiographs from the previous admission did not show any evidence of a pneumatocele or pneumothorax, which were noted on the second admission indicating that the pneumatocele was indeed a new lesion. In cases one and three, on the other hand, no previous chest X-rays were available for comparison. No change in the appearance of the pneumatoceles was noted with pleural cavity drainage.

Our review of the literature also revealed a case report of a COVID-19 patient from Wuhan, China who had not been on invasive mechanical ventilation developing spontaneous pneumothorax, pneumomediastinum, and a giant bulla in the left lung [5]. This patient had serial CT scans, which showed mediastinal emphysema on day 11 of admission (at which time he also developed a high fever) and a giant bulla in the left lung on day 26 of admission. On day 34, a left pneumothorax and pleural effusion were evident. While levels of inflammatory markers are not mentioned in this article, we again note a similar pattern of development of these lesions after a certain duration of illness. To our knowledge, autopsies were not performed for the patients presented in this article. Nevertheless, we did review the literature on autopsy findings in COVID-19 patients which indicates that pulmonary pathologic findings reported are predominantly those of diffuse alveolar damage which are not only similar to those in SARS patients but also support the hypothesis of pulmonary parenchymal injury leading to the development of pneumothorax [6, 7]. Other findings include acute bronchopneumonia [6] and pulmonary embolism [7]. A review of CT findings noted in patients in Wuhan, China showed bilateral ground-glass infiltrates to be the predominant features [8]. A systematic review of CT findings in 919 patients showed similar findings in addition to less common features, including pneumothoraces and lung cavitation [9]. While bilateral lung infiltrates are the most commonly reported radiologic features in COVID-19 pneumonia pneumothorax, pneumomediastinum and cavitary lung lesions do obviously occur, albeit less commonly. 

Finally, we note that the management of these lesions with tube thoracostomy or even observation provided satisfactory outcomes. Two of our three patients were discharged home and while one patient did die, this was due to the development of multiorgan dysfunction rather than due to inadequate management of the pneumothorax. Operative management of pneumothoraces in COVID-19 patients has been described with bedside thoracoscopic blebectomy and pleurodesis [10]. This approach definitely has its merits, including a quicker resolution of air leaks and, therefore, shorter time to chest tube removal and discharge from the hospital. However, we feel that outcomes in COVID-19 pneumonia depend more on the severity of the underlying lung disease and impairment of gas exchange and therefore non-operative management of pneumothoraces in these patients also provides adequate outcomes although the length of stay may be longer (25 and 21 days in cases two and three respectively). 

We believe that the combination of severe inflammation and prolonged duration of illness in COVID-19 patients gives rise to pneumothorax, pneumomediastinum, and pneumatoceles due to degenerative changes in the lung parenchyma. At this point, it is not clear why only one percent of patients with COVID-19 pneumonia develop pneumothorax. Other factors are most likely needed for the development of pneumothorax, and these may become clearer as we continue to move through this pandemic. Managing pneumothoraces in these patients is likely crucial to prevent the development of life-threatening tension pneumothoraces. While operative management in these patients is definitely an option, it seems that management with chest tubes or even observation provides satisfactory outcomes, although a prolonged duration of stay may result in non-operative management due to the time needed for resolution of air leaks from injured and fibrotic pulmonary parenchyma. 

## Conclusions

Pneumothorax, pneumomediastinum and cystic lung lesions such as pneumatoceles in COVID-19 patients likely result from prolonged inflammatory damage to lung parenchyma with development of degenerative changes and subsequent air leaks. This indeed appears similar to the pathogenesis of pneumothoraces in SARS which also is caused by a virus from the same *Coronaviridae* family.

Pleural cavity decompression may help prevent development of life-threatening tension pneumothoraces while more invasive surgical management may be considered help decrease time to chest tube removal and length of stay, factors which may be critical in overloaded health systems trying to make beds available to incoming critically ill patients. Nevertheless, we believe that the final outcome of the patient depends on the progression of the underlying pulmonary parenchymal disease, the trajectory of which would not be altered by operative management of pneumothoraces.
